# Modified future diurnal variability of the global surface ocean CO_2_ system

**DOI:** 10.1111/gcb.16514

**Published:** 2022-11-20

**Authors:** Lester Kwiatkowski, Olivier Torres, Olivier Aumont, James C. Orr

**Affiliations:** ^1^ LOCEAN Laboratory Sorbonne Université‐CNRS‐IRD‐MNHN Paris France; ^2^ LMD‐IPSL, CNRS, Ecole Normale Supérieure/PSL Research University, Ecole Polytechnique Sorbonne Université Paris France; ^3^ Laboratoire des Sciences du Climat et de l'Environnement, LSCE‐IPSL, CEA‐CNRS‐UVSQ Université Paris Saclay Gif‐sur‐Yvette France

**Keywords:** climate change, CO_2_, diel, diurnal, marine carbonate chemistry, ocean acidification

## Abstract

Our understanding of how increasing atmospheric CO_2_ and climate change influences the marine CO_2_ system and in turn ecosystems has increasingly focused on perturbations to carbonate chemistry variability. This variability can affect ocean‐climate feedbacks and has been shown to influence marine ecosystems. The seasonal variability of the ocean CO_2_ system has already changed, with enhanced seasonal variations in the surface ocean *p*CO_2_ over recent decades and further amplification projected by models over the 21st century. Mesocosm studies and CO_2_ vent sites indicate that diurnal variability of the CO_2_ system, the amplitude of which in extreme events can exceed that of mean seasonal variability, is also likely to be altered by climate change. Here, we modified a global ocean biogeochemical model to resolve physically and biologically driven diurnal variability of the ocean CO_2_ system. Forcing the model with 3‐h atmospheric outputs derived from an Earth system model, we explore how surface ocean diurnal variability responds to historical changes and project how it changes under two contrasting 21st‐century emission scenarios. Compared to preindustrial values, the global mean diurnal amplitude of *p*CO_2_ increases by 4.8 μatm (+226%) in the high‐emission scenario but only 1.2 μatm (+55%) in the high‐mitigation scenario. The probability of extreme diurnal amplitudes of *p*CO_2_ and [H^+^] is also affected, with 30‐ to 60‐fold increases relative to the preindustrial under high 21st‐century emissions. The main driver of heightened *p*CO_2_ diurnal variability is the enhanced sensitivity of *p*CO_2_ to changes in temperature as the ocean absorbs atmospheric CO_2_. Our projections suggest that organisms in the future ocean will be exposed to enhanced diurnal variability in *p*CO_2_ and [H^+^], with likely increases in the associated metabolic cost that such variability imposes.

## Graphical abstract

Using a global ocean biogeochemical model, we project how diurnal variability of the surface ocean CO_2_ system responds to historical and future projections of climate change. In a high 21st‐century emission scenario, the global mean diurnal amplitude of *p*CO_2_ increases threefold compared to preindustrial values, with a dramatic increase in the probability of extreme diurnal events. The main driver of heightened *p*CO_2_ diurnal variability in the surface open ocean is the enhanced sensitivity of *p*CO_2_ to changes in temperature as the ocean absorbs anthropogenic carbon.

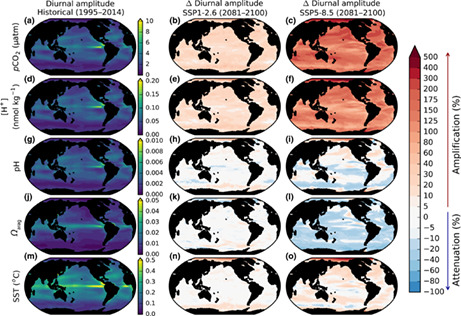

## 1 INTRODUCTION

Substantial oceanic uptake of anthropogenic carbon from the atmosphere has been identified since the mid‐20th century (Revelle & Suess, 1957), with the aggregate impact on the marine CO_2_ system becoming gradually termed “ocean acidification” (Brewer, 2013). Seasonal and higher temporal frequency variations of the ocean CO_2_ system driven by physical and biological processes have been characterized, at local scales, since the late 1970s (Codispoti et al., 1982; Feely et al., 1988; Johnson et al., 1979; Simpson & Zirino, 1980; Weiss et al., 1982). However, early model projections of 21st century ocean carbonate chemistry under different emission scenarios focused on annual mean changes (Kleypas et al., 1999), with subannual variability estimated as negligible outside the high latitudes (Orr et al., 2005). Since then there has been growing interest in how the subannual temporal variability of the ocean CO_2_ system responds to ocean carbon uptake and climate change. This has been partly motivated by the recognition that seasonal variability affects ocean extreme events (e.g., earlier incidence of calcium carbonate undersaturation; Gruber et al., 2012; McNeil & Matear, 2008; Sasse et al., 2015) and that such variability is itself sensitive to anthropogenic perturbations of the climate system (Burger et al., 2020; Kwiatkowski & Orr, 2018; McNeil & Sasse, 2016).

Observational products indicate that the global average amplitude of the seasonal cycle in surface open ocean *p*CO_2_ increased by 2.2 μatm per decade between 1982 and 2015 (Landschützer et al., 2018). Such amplification is consistent with Earth system models, which project 1.5‐ to 3‐fold increases in the amplitude of the seasonal cycle of surface ocean *p*CO_2_ under high 21st‐century emission scenarios (Gallego et al., 2018; McNeil & Sasse, 2016). Similar amplification of the seasonal cycle of the hydrogen ion concentration [H^+^], which is near‐linearly related to *p*CO_2_, is likewise projected under high‐emission pathways, while models generally project relatively minor attenuation of the seasonal cycles of pH (−10% to 16%) and *Ω*_arag_ (−9%) (Kwiatkowski et al., 2020; Kwiatkowski & Orr, 2018). These seasonal perturbations are primarily the result of ocean carbon uptake altering the sensitivity of CO_2_ system variables to changes in temperature, dissolved inorganic carbon (DIC), and alkalinity (Fassbender et al., 2018; Gallego et al., 2018; Kwiatkowski & Orr, 2018). Additionally, physical climate change also drives regional trends in the seasonal amplitudes of these drivers.

Alongside changes to seasonal cycles, diurnal cycles of the ocean CO_2_ system variables are also expected to respond to rising atmospheric CO_2_ and climate change. However, robust historical trends cannot be detected in the short observational records of diurnal cycles (<14 years), which require high‐frequency monitoring (Torres et al., 2021). Nonetheless, observations from natural CO_2_ vent sites indicate that at higher background DIC concentrations, the diurnal amplitude of *p*CO_2_ and [H^+^] is enhanced while that of *Ω*_arag_ is attenuated (Kerrison et al., 2011; Kwiatkowski & Orr, 2018). Mesocosm experiments have also shown that when the ocean fugacity of CO_2_ is increased from 310 to 675 μatm, roughly the same diurnal cycle of DIC, which is driven by net community production, produces a tripling in the amplitudes of *p*CO_2_ and [H^+^] diurnal cycles (Schulz & Riebesell, 2013). Furthermore, simple one‐dimensional models of habitat‐specific CO_2_ system variability, project enhanced diurnal cycles of *p*CO_2_ and attenuated cycles of *Ω*_arag_ under a moderately high‐emission pathway (Takeshita et al., 2015). All of these studies have accounted for how increased DIC concentrations affect diurnal variability of the ocean CO_2_ system by reducing ocean buffer capacity (Egleston et al., 2010; Frankignoulle, 1994). However, coincident changes in the diurnal cycles of the principal controlling variables of the CO_2_ system are neglected. In addition, past studies have not accounted for how the probability and magnitude of extreme diurnal cycles (particularly high peak‐to‐peak diurnal amplitudes) of the ocean CO_2_ system may respond to climate change. Extreme diurnal cycles are generally thermally driven in the open ocean but biologically driven in coastal environments (Torres et al., 2021). The magnitude of such extreme diurnal cycles can exceed that of seasonal cycles in both the open and coastal oceans, resulting in rapidly changing conditions that may affect ecosystems (Bates et al., 1998; Torres et al., 2021).

The influence of CO_2_ system temporal variability on organism physiology is complex and varied. Experimental studies on ocean acidification have typically held ambient seawater chemistry conditions at differing fixed values to explore organism sensitivities (Boyd et al., 2016; Kroeker et al., 2020). When diurnal variability of the seawater CO_2_ system has also been accounted for, there are highly diverse impacts on energetic costs, growth, and the tolerance of species to acidification (Alenius & Munguia, 2012; Bitter et al., 2021; Cornwall et al., 2013; Dufault et al., 2012; Jarrold & Munday, 2018; Jiang et al., 2019; Kapsenberg et al., 2018; Laubenstein et al., 2020; Rivest et al., 2017). Given that both species and ecosystems (e.g., Albright et al., 2016, 2018) are sensitive to subdaily variability of carbonate chemistry, understanding how diurnal cycles of the ocean CO_2_ system will respond to climate change may be critical to projecting biological impacts.

Here, we perform multi‐centennial simulations with a global ocean biogeochemical model that includes ocean physics, biogeochemistry, and sea ice, and has been adapted to resolve diurnal cycles of marine net primary production (NPP). The model is forced with 3‐h atmospheric fields derived from past simulations of a fully coupled Earth system model, which, in addition to ocean physics, biogeochemistry, and sea ice, also contains an atmospheric general circulation model and land surface/terrestrial biosphere model. This combination permits the global ocean biogeochemical model to simulate the diurnal variability of the surface ocean CO_2_ system under preindustrial conditions, over the industrial era and in two scenarios of 21st century climate change. Our aim is to address the following questions: (i) How are mean and extreme diurnal cycles of the ocean CO_2_ system likely to respond to rising atmospheric CO_2_ and associated climate change? (ii) What are the physical and biogeochemical drivers of these changes and how do they manifest regionally? (iii) How does accounting for increased diurnal variability of key ocean CO_2_ system variables influence the probability that ecosystems are exposed to conditions that may impact organism fitness?

## 2 MATERIALS AND METHODS

To evaluate how diurnal cycles of the CO_2_ system are influenced by ocean acidification and climate change, we use the NEMO version 4.0 ocean modelling platform (Madec et al., 2019). Ocean dynamics and thermodynamics in NEMO are based on the legacy of the ocean dynamical core OPA (Madec et al., 1998), coupled to the SI^3^ sea ice model and the PISCES‐QUOTA biogeochemical model. PISCES‐QUOTA (Kwiatkowski et al., 2018) is a variable phytoplankton stoichiometry version of the PISCES model (Aumont et al., 2015). It simulates the planktonic trophic levels of marine ecosystems as well as the biogeochemical cycles of carbon and key ocean nutrients (nitrate, ammonium, phosphate, silicate, and iron). In total, it has 39 prognostic tracers including three phytoplankton size classes/groups (picophytoplankton, nanophytoplankton, and diatoms) and two zooplankton size classes (microzooplankton and mesozooplankton). The ocean CO_2_ system in PISCES‐QUOTA follows the Ocean Model Intercomparison Project (OMIP) protocols (Orr et al., 2017), with DIC and total alkalinity represented as volumetric prognostic tracers. The ocean exchanges CO_2_ with the atmosphere at the sea surface with the instantaneous gas transfer velocity computed as a quadratic function of 10‐m wind speeds according to the relationship of Wanninkhof (1992). Alongside the influence of physical changes in temperature, salinity, and gas exchange, multiple biological processes influence the simulated ocean CO_2_ system on subdaily timescales in the upper ocean (Kwiatkowski et al., 2018). These processes include heterotrophic respiration of zooplankton, the remineralization of dissolved organic matter by implicit bacteria, and the dissolution of CaCO_3_. PISCES‐QUOTA typically computes phytoplankton NPP and implicit calcification at daily temporal resolution, but in this study, this was modified to 3‐h time steps synchronous with the atmospheric forcing inputs. This modification also allows subdaily fluxes of DIC associated with the respiratory cost of phytoplankton uptake of ammonium (possible during the day and night) and nitrate (only possible during the day).

### 2.1 Simulations

The NEMO‐PISCES‐QUOTA model was run on an ORCA tripolar global configuration with a nominal horizontal resolution of 1° and meridional refinement of up to 1/3° at the equator (ORCA1). The model configuration is discretized into 75 *z*‐coordinate vertical levels with thicknesses increasing from 1 m in the surface ocean to a maximum of 204 m in the abyssal ocean. The model was forced with 3‐h atmospheric outputs (near surface wind speed, air temperature, specific humidity, surface downwelling shortwave and longwave radiation, precipitation, and surface air pressure) derived from concentration‐driven simulations of the IPSL‐CM6‐LR Earth system model (Boucher et al., 2020) used in the recent Coupled model Intercomparison Project Phase 6 (CMIP6, Eyring et al., 2016; O'Neill et al., 2016). In addition to ocean physics, sea ice, and a reduced complexity ocean biogeochemistry component, IPSL‐CM6‐LR also includes an atmospheric general circulation model and land surface and terrestrial biosphere model. In total, four NEMO‐PISCES‐QUOTA simulations were performed:

- a preindustrial control simulation of 295 years used to run the model to a quasi‐equilibrium state and assess diurnal CO_2_ system variability prior to anthropogenic influence on the ocean‐climate system;
- a historical simulation during 1850–2014;
- a 2015–2100 simulation under the SSP1‐2.6 low‐emission scenario (Meinshausen et al., 2020); and
- a 2015–2100 simulation under the SSP5‐8.5 high‐emission scenario (Riahi et al., 2017).

Simulations were forced with atmospheric output derived from the respective picontrol, historical, SSP1‐2.6, and SSP5‐8.5 IPSL‐CM6‐LR simulations (Boucher et al., 2020; Lurton et al., 2020) produced for CMIP6 (Eyring et al., 2016; O'Neill et al., 2016). The atmospheric CO_2_ concentration was an annually prescribed global value, as in IPSL‐CM6‐LR, in accordance with CMIP6 guidelines (Meinshausen et al., 2017, 2020). In the preindustrial control, it is held constant at 284 ppm, and in the historical run, it rises from 284 to 398 ppm between 1850 and 2014. In SSP1‐2.6, it peaks at 474 ppm in 2063–2064 and then declines to 446 ppm in 2100, while in SSP5‐8.5, it increases throughout the 21st century reaching 1135 ppm in 2100.

Surface ocean CO_2_ system outputs were calculated from 3‐h model output over the duration of simulations. That is, the 3‐h NEMO‐PISCES‐QUOTA simulated fields of surface ocean DIC, total alkalinity, phosphate, silicate, sea surface temperature (SST), and salinity were used to compute surface ocean CO_2_ system variables offline with the *mocsy* software package (Orr & Epitalon, 2015).

### 2.2 Model validation

The modified NEMO‐PISCES‐QUOTA model was validated relative to present‐day, annual‐mean, surface‐ocean conditions, with simulated fields compared to observations of SST (Locarnini et al., 2018), salinity (Zweng et al., 2019), nutrient concentrations (Garcia et al., 2019; Locarnini et al., 2018; Zweng et al., 2019), DIC and total alkalinity (Lauvset et al., 2016), *p*CO_2_ (Landschützer et al., 2020), chlorophyll (Hu et al., 2012; Werdell & Bailey, 2005), and NPP (Behrenfeld et al., 2005; Westberry et al., 2008). The simulated diurnal variability of the CO_2_ system was compared to recent observational diurnal analyses at open ocean and coastal stations where multi‐annual, high‐resolution (3‐h) observations of the CO_2_ system exist (Sutton et al., 2019; Torres et al., 2021).

### 2.3 Analysis of projections

The peak‐to‐peak diurnal amplitude of ocean CO_2_ system variables was calculated as the difference between maximum and minimum 3‐h outputs for each 24‐h day. The peak‐to‐peak seasonal amplitude was calculated as the difference between maximum and minimum monthly mean outputs for each year. All regional and global means of ocean CO_2_ system metrics represent area‐weighted means of metrics that are calculated on the native NEMO grid.

### 2.4 The probability of extreme diurnal events

To assess how extreme diurnal amplitudes respond to anthropogenic climate change, we define extremes locally relative to a preindustrial baseline. In total, 245 years of 3‐h model output under preindustrial forcing were used to characterize the natural variability of the diurnal amplitude of the CO_2_ system. This represents our fixed baseline period and was used to compute changes in the probability of extreme diurnal amplitudes. For each grid cell, the 99th and 99.9th percentiles of CO_2_ system diurnal amplitudes were calculated over this 245‐year preindustrial period. The frequency that these percentiles are exceeded in historical and SSP simulations was similarly calculated. Changes in the probability of extreme diurnal amplitudes are expressed as probability ratios (Pr) in line with approaches that have been applied to studies of marine heat waves (Frölicher et al., 2018). Pr are defined, at each grid cell, as the probability of a given threshold being exceeded in a period of the historical or SSP simulations (Pi) relative to the probability of that threshold being exceeded in the preindustrial control (Pc): Pr = Pi/Pc. A Pr of 10 therefore represents a 10‐fold increase in the probability of a given extreme diurnal amplitude occurring.

### 2.5 Decomposing the drivers of diurnal variability

In order to assess the thermal and nonthermal drivers of diurnal *p*CO_2_ system variability, mean diurnal cycles of DIC (*C*_T_), total alkalinity (*A*_T_), temperature (*T*), salinity (*S*), phosphate, and silicate were computed for the surface ocean using 3‐h model output for the last 20 years of both the historical simulation (1995–2014) and SSP5‐8.5 (2081–2100). For each grid cell in the two 20‐year periods, we distinguished contributions to the mean diurnal cycle of *p*CO_2_ with a Taylor‐series deconvolution (Takahashi, 1993):

(1) $$\Delta p{\mathrm{CO}}_{2}=\underset{\text{Thermal component}}{\underbrace{\left(\frac{\partial p{\mathrm{CO}}_{2}}{\partial T}\right)\Delta T}}+\underset{\text{Nonthermal component}}{\underbrace{\left(\frac{\partial p{\mathrm{CO}}_{2}}{\partial C_{T}}\right)\Delta C_{T}+\left(\frac{\partial p{\mathrm{CO}}_{2}}{\partial A_{T}}\right)\Delta A_{T}+\left(\frac{\partial p{\mathrm{CO}}_{2}}{\partial S}\right)\Delta S}}$$

where *Δ* terms are diurnal anomalies relative to the daily mean and the partial differentials (sensitivities) are estimated numerically from the mean diurnal cycle of absolute values of *C*_T_, *A*_T_, *T*, *S*, phosphate, and silicate using *mocsy* (Orr & Epitalon, 2015). As shown above, the deconvolution can be separated into (i) a thermal component that is the product of the thermal sensitivity of a CO_2_ system variable and the temperature anomaly associated with a diurnal time step and (ii) a nonthermal component that is the sum of the three other deconvolution terms. Thus, the diurnal variability of surface ocean *p*CO_2_ was partitioned into thermally and nonthermally driven contributions at the grid‐cell scale for both the recent past and the projected end‐of‐century conditions under the high‐emission scenario SSP5‐8.5. The performance of the deconvolution was assessed by comparing the sum of its four terms to the mean diurnal cycle of *p*CO_2_ anomalies actually simulated by NEMO‐PISCES‐QUOTA and found to differ by typically <1%.

## 3 RESULTS

### 3.1 Model evaluation

#### 3.1.1 Mean physical and biogeochemical state

The annual mean state of physical and biogeochemical surface ocean fields simulated by NEMO‐PISCES‐QUOTA is similar to observations (Figures S1 and S2). Meridional and zonal patterns in SST and salinity are generally well represented, as are the spatial distributions of surface ocean nutrient (Si, NO_3_^−^) concentrations. The model broadly captures spatial patterns in DIC and alkalinity in the surface ocean. Although it overestimates both, spatial patterns of mean surface ocean *p*CO_2_ are globally well produced, albeit with slight negative biases in the subtropical southern hemisphere and Arctic, and slight positive biases in the subtropical northern hemisphere and certain parts of the Southern Ocean. Simulated global mean, depth integrated, NPP also compares favorably to observations, with high productivity in tropical regions, low productivity in subtropical gyres, and reasonable values in the major high‐nutrient, low‐chlorophyll regions.

#### 3.1.2 Diurnal CO_2_ system variability

**FIGURE 1:**
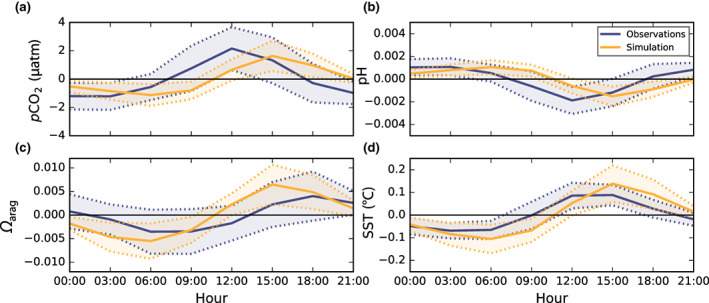
Evaluation of simulated diurnal cycles of the CO_2_ system in the surface open ocean against observations. Mean simulated (orange) and observed (blue; Sutton et al., 2019; Torres et al., 2021) diurnal cycles of (a) *p*CO_2_, (b) pH, (c) *Ω*_arag_, and (d) temperature across 15 open ocean stations, with model outputs extracted from the nearest grid cell to each observational station over 1995–2014. Due to the averaging of nonsynchronous diurnal cycles across multiple stations/model grid cells, the simulated mean diurnal cycle amplitudes shown here are reduced relative to the values given in Figure 2, and more so for the observations, which differ more in phasing between stations (Figures S3 and S4).

Long‐term observations of diurnal physical and biogeochemical variability in the surface ocean are sparse, being typically associated with specific moored observational platforms. In order to evaluate model performance at capturing diurnal variability, we use multiple in situ autonomous *p*CO_2_ and pH time series for which the CO_2_ system and its associated diurnal and seasonal variability have been characterized previously (Sutton et al., 2016, 2019; Torres et al., 2021). At open ocean stations (bottom depths >1000 m) where multi‐annual, 3‐h observations exist, the model broadly captures the phasing of diurnal anomalies of the CO_2_ system (Figure 1). Both observed and simulated diurnal maxima of SST, *p*CO_2_, and [H^+^] occur in early to mid‐afternoon, while their minima occur toward the end of the night. At the same open ocean stations, the observed diurnal cycle of *p*CO_2_ has been previously shown to be driven by SST variability and moderated slightly by diurnal changes in DIC (Torres et al., 2021). The model simulates similar thermal dominance of diurnal *p*CO_2_ variability given that non‐thermal processes (i.e., NPP) would act to produce negative *p*CO_2_ anomalies during the day and positive *p*CO_2_ anomalies at night. The simulated diurnal cycle of *Ω*_arag_ is also driven by temperature variability with maximum *Ω*_arag_ values at 15:00 and minimum values at 06:00 coincident with maximum and minimum SST anomalies. However, observations show greater diversity in the timing of diurnal *Ω*_arag_ cycles across stations (compare Figures S3 and S4). Although some observational stations show diurnal *Ω*_arag_ maxima at 15:00, most exhibit maxima at 18:00 when diurnal SST anomalies are slightly lower (although still positive). Thus, nonthermal processes may play a greater and more variable role in observed diurnal cycles of *Ω*_arag_.

**FIGURE 2:**
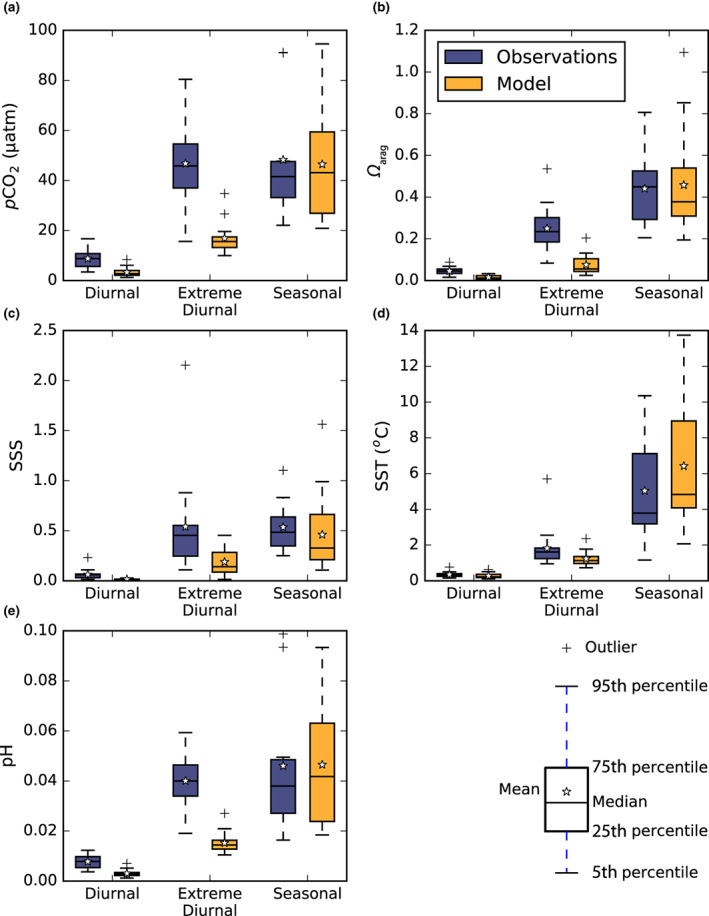
Evaluation of the amplitude of simulated diurnal, extreme diurnal, and seasonal cycles of the CO_2_ system in the surface open ocean against observations. The modeled (orange) open ocean diurnal, extreme diurnal, and seasonal cycles of (a) *p*CO_2_, (b) *Ω*_arag_, (c) salinity, (d) temperature, and (e) pH compared to fixed time‐series observations (blue; Sutton et al., 2019; Torres et al., 2021). Box plots are produced from the station mean amplitude of diurnal, extreme diurnal, and seasonal cycles across 15 open ocean observational stations with model outputs extracted from the nearest grid cell to each observational station over the period 1995–2014. Extreme diurnal cycles represent 99th percentile diurnal amplitudes.

Although the model roughly matches the observed seasonal amplitudes of surface ocean temperature, salinity, *p*CO_2_, pH, and *Ω*_arag_, it underestimates corresponding diurnal amplitudes (Figure 2). This underestimation could have multiple causes, including but not limited to (i) biases in the prescribed surface fluxes derived from the IPSL‐CM6‐LR Earth system model, (ii) influence of the ocean skin layer and mixing processes unresolved by the model, and (iii) overestimated diurnal cycles of NPP in these regions. Although the model underestimates the observed magnitudes of diurnal and extreme (99th percentile) diurnal cycles in the present‐day open ocean, the relative increase in the amplitude of an extreme diurnal cycle compared to the mean diurnal cycle is similar. Both have a 5.5‐fold increase on average.

Furthermore, despite the model's apparent underestimation of diurnal CO_2_ system variability in the open ocean, the mechanisms underpinning this variability appear consistent with observations. That is, diurnal *p*CO_2_ variability is driven by diurnal changes in temperature and offset partly by combined nonthermal effects. This consistency is not the case at coastal ocean stations (bottom depths 16–780 m), where despite the model being in phase with observed diurnal SST variability, it is in antiphase with observed diurnal cycles of *p*CO_2_, pH, and *Ω*_arag_, and it has a much larger negative bias with respect to simulated diurnal amplitudes (Figures S5 and S6). The poor model performance in the coastal ocean is consistent with expectations. Diurnal CO_2_ system variability in the coastal ocean observations is typically not dominated by thermal effects but by DIC variations (Torres et al., 2021), and that is often heavily influenced by productive biological systems such as kelp forests, seagrasses, and coral reefs (Berg et al., 2019; Drupp et al., 2013; Hofmann et al., 2011; Murie & Bourdeau, 2020), all of which are absent in the model. In addition, our global model does not resolve tidal oscillations and is thus unlikely to adequately represent water residence times in the coastal ocean, another critical determinant of CO_2_ system variability on diurnal timescales (Page et al., 2019). In summary, the model fails in the coastal ocean, but in the open ocean, the phasing of simulated diurnal cycles of the CO_2_ system compares reasonably well with observations, albeit with a general underestimation of diurnal amplitudes. The dominance of the thermal component for open ocean diurnal variability in both the model and observations supports use of the model to assess potential changes in that variability.

### 3.2 Model projections

#### 3.2.1 Interannual, seasonal, and diurnal changes to the ocean CO_2_ system

The global annual mean surface ocean *p*CO_2_ simulated by NEMO‐PISCES‐QUOTA increases from 284 to 383 μatm between 1850 and 2014 (the historical simulation period). Under the high‐emission scenario SSP5‐8.5, global annual mean surface ocean *p*CO_2_ is projected to increase to 1090 μatm by 2100. In contrast under the high mitigation scenario SSP1‐2.6, it reaches a maximum of 462 μatm in 2063 and decreases thereafter to 439 μatm by 2100. Annual mean surface ocean pH decreases from 8.17 in 1850 to 8.06 at the end of the historical period, a reduction that is consistent with observed pH decline of 0.018 per decade between 1991 and 2011 (Lauvset et al., 2015). By 2100, pH declines further to 7.99 under SSP1‐2.6 and to 7.66 under SSP5‐8.5. These values are consistent with CMIP6 multi‐model mean earth system model simulations (Kwiatkowski et al., 2020). Alongside increasing *p*CO_2_ and declining pH, the global‐average, annual‐mean *Ω*_arag_ decreases from 3.42 in 1850 to 2.94 at the end of the historical period. Under SSP5‐8.5, it declines to 1.61 by 2100, while under SSP1‐2.6, it reaches a minimum of 2.67 in 2061 and then increases to 2.76 in 2100.

**FIGURE 3:**
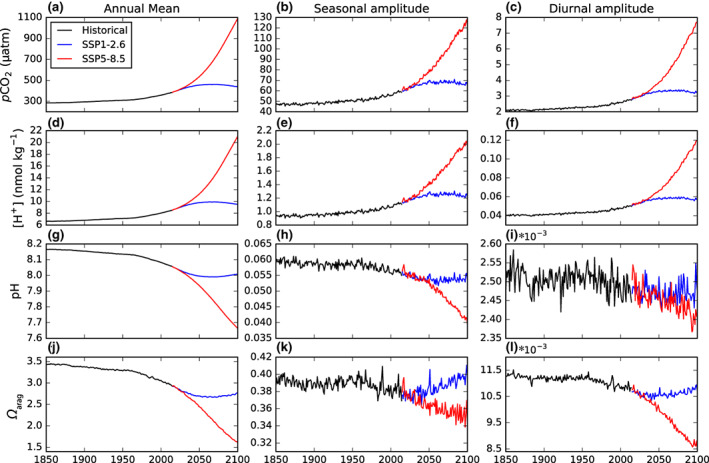
Global‐average changes in the annual mean, seasonal amplitude, and diurnal amplitude of (a–c) *p*CO_2_, (d–f) [H^+^], (g–i) pH, and (j–l) *Ω*_arag_ over the historical and the two SSP simulations. All values are calculated at the grid‐cell scale and area‐weighted to give global surface ocean means. Amplitudes are peak‐to‐peak values calculated as the seasonal or diurnal maximum minus minimum.

Over the historical simulation, the global mean peak‐to‐peak seasonal amplitude of ocean *p*CO_2_ increases from 47 μatm (1850–1869) to 57 μatm (1995–2014), with most of this 21% increase occurring in the late 20th and early 21st century (Figure 3; Table S1). This increase is consistent with observations of a 2.2 μatm increase per decade between 1982 and 2015 (Landschützer et al., 2018). By 2081–2100, the seasonal amplitude of ocean *p*CO_2_ is projected to increase to 67 μatm under SSP1‐2.6 and 117 μatm under SSP5‐8.5. Compared to preindustrial values, these increases represent seasonal amplifications of 44% under SSP1‐2.6 and 151% under SSP5‐8.5. This amplification is consistent with past projections from Earth system models and results mainly from heightened background DIC concentrations increasing the sensitivity of ocean *p*CO_2_ to seasonal variations in DIC and temperature (Gallego et al., 2018; McNeil & Sasse, 2016).

Alongside the amplification of the seasonal cycle of ocean *p*CO_2_, the seasonal amplitude of pH is projected to attenuate by 0.003 (−5%) by the end of the historical simulation, 0.005 (−8%) by the end of SSP1‐2.6, and 0.016 (−27%) by the end of SSP5‐8.5 (Figure 3; Table S1). Given that ocean [H^+^] is near‐linearly related to *p*CO_2_ on annual timescales (Orr, 2011), the attenuation of pH seasonal cycles, although counterintuitive, is a consequence of the log transformation of [H^+^] in pH (Fassbender et al., 2021; Kwiatkowski et al., 2020; Kwiatkowski & Orr, 2018). It follows that the seasonal amplitude of pH is proportional to the seasonal amplitude of [H^+^] and inversely proportional to the annual mean [H^+^]. Under high‐emission scenarios, most models (including NEMO‐PISCES‐QUOTA) project a greater relative increase in annual mean [H^+^] than in the seasonal amplitude of [H^+^], and thus, the seasonal amplitude of pH is attenuated (Kwiatkowski et al., 2020; Kwiatkowski & Orr, 2018). Our projected attenuation of the pH seasonal amplitude is comparable to the CMIP6 multi‐model attenuation of −10 ± 5% under SSP5‐8.5 (2080–2099 relative to 1995–2014; Kwiatkowski et al., 2020) and the CMIP5 multi‐model attenuation of −16 ± 7% under RCP8.5 (2090–2099 relative to 1990–1999; Kwiatkowski & Orr, 2018).

Along with amplification of *p*CO_2_ seasonal cycles and attenuation of pH seasonal cycles, the model also projects scenario‐dependent changes in the global mean seasonal amplitude of *Ω*_arag_. Over the historical period, the *Ω*_arag_ seasonal amplitude attenuates by 0.001 (−3%) while also showing high interannual and multi‐decadal variability (Figure 3). Under SSP1‐2.6, the seasonal amplitude of *Ω*_arag_ rebounds to early historical values, while under SSP5‐8.5, it attenuates by 0.004 (−10%). That reduction under high emissions is much like the −9 ± 8% attenuation seen for the CMIP5 multi‐model mean under RCP8.5 (2090–2099 relative to 1990–1999; Kwiatkowski & Orr, 2018). Attenuation of *Ω*_arag_ seasonal amplitude has been shown, like the amplification of *p*CO_2_ and [H^+^] seasonal amplitudes, to be predominately driven by the geochemical effect of increasing background DIC. For *Ω*_arag_, increasing DIC decreases its sensitivity to changes in DIC, although in subtropical regions, this reduced sensitivity can more than compensated by increases in the seasonal amplitude of DIC, and thus increase the seasonal amplitude of *Ω*_arag_ regionally (Kwiatkowski & Orr, 2018).

The global mean diurnal amplitudes of *p*CO_2_, *Ω*_arag_, and to a lesser extent pH exhibit similar trends of amplification and attenuation to those observed for the seasonal amplitudes. The diurnal amplitude of *p*CO_2_ in the surface ocean increases, on average, from 2.1 μatm (1850–1869) to 2.7 μatm (1995–2015) (+26%) over the historical simulation. By the end of the 21st century, the global average diurnal amplitude of *p*CO_2_ is projected to increase to 3.3 μatm under SSP1‐2.6 and 6.9 μatm under SSP5‐8.5. Relative to 1850–1869 values, this represents an increase in the diurnal amplitude of +55% and +226% under SSP1‐2.6 and SSP5‐8.5, respectively. The global mean diurnal amplitude of *Ω*_arag_ exhibits much smaller relative changes, declining by 0.0004 (−4%) over the historical period. By the final 20 years of SSP1‐2.6, the decrease in the *Ω*_arag_ diurnal amplitude is 0.0006 (−5%), while under SSP5‐8.5, it reaches 0.0025 (−22%). Trends in the diurnal amplitude of pH are limited, particularly in the historical and SSP1‐2.6 simulations. There is, however, a slight decline in the diurnal amplitude of pH in SSP5‐8.5 (−4%).

Due to the pH log scale, trends in the diurnal amplitude of pH represent the ratio of the diurnal amplitude in [H^+^] to the diurnal mean [H^+^]. Relative to 1850–1869 historical values, the diurnal amplitude of [H^+^] is amplified 23% by 1995–2014, 45% by 2081–2100 under SSP1‐2.6, and 170% by 2081–2100 under SSP5‐8.5. These diurnal increases in [H^+^] are slightly lower than those of *p*CO_2_, consistent with the near‐linear relationship between *p*CO_2_ and [H^+^] for long‐term trends (Orr, 2011) and for changes in seasonal variations (Kwiatkowski & Orr, 2018).

#### 3.2.2 Spatial differences in altered diurnal cycles

**FIGURE 4:**
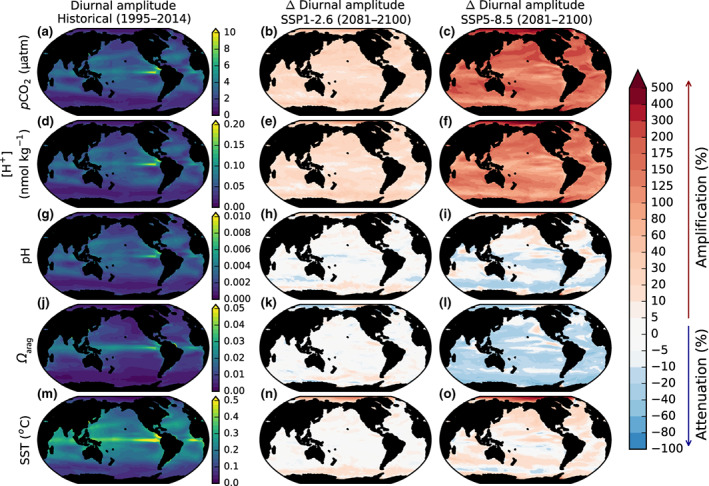
The simulated mean diurnal amplitudes of *p*CO_2_, [H^+^], pH, *Ω*_arag_, and SST and their changes under SSP1‐2.6 and SSP5‐8.5. The simulated mean modern (1995–2014) diurnal amplitudes of *p*CO_2_, [H^+^], pH, *Ω*_arag_, and SST and their change in 2081–2100 under SSP1‐2.6 and SSP5‐8.5 relative to the historical period.

The simulated diurnal amplitudes of ocean CO_2_ system variables (1995–2014 mean values) show high spatial variability (Figure 4). The dominant features of this variability are (i) strong meridional gradients in the simulated diurnal amplitudes of *p*CO_2_, pH, and *Ω*_arag_, with enhanced amplitudes in the lower latitudes and reduced amplitudes in the higher latitudes, and (ii) enhanced diurnal amplitudes of *p*CO_2_, pH, and *Ω*_arag_ in coastal regions and the eastern equatorial upwelling systems of the Pacific and Atlantic basins. These features are mainly attributable to patterns in the simulated diurnal amplitude of SST, the principal driver of diurnal variability in the surface ocean CO_2_ system in the model.

The diurnal amplitude of *p*CO_2_ is projected to amplify globally under both SSP1‐2.6 and SSP5‐8.5, each with similar amplification everywhere except in the Arctic where it is substantially larger (Figure 4; Figure S7). This regional enhancement may result from the weak diurnal amplitude of *p*CO_2_ in the Arctic toward the end of the historical period when permanent sea ice cover is high, combined with the progressive loss of sea ice over the SSPs, which dramatically amplifies the diurnal amplitude of SST.

In SSP1‐2.6, the diurnal amplitude of pH exhibits little change compared to the end of the historical simulation, except for parts of the Arctic Ocean where some amplification is projected. Under SSP5‐8.5, enhanced amplification of the diurnal amplitude of pH is projected in the Arctic Ocean as well as the Weddell Sea; however, there is attenuation elsewhere, notably in the tropical Pacific and in other regions of the Southern Ocean. Relative changes in the diurnal amplitude of *Ω*_arag_ are limited under SSP1‐2.6, with most ocean basins exhibiting slight amplification and attenuation in different areas. Under SSP5‐8.5, however, nearly all ocean basins exhibit attenuation of the diurnal amplitude of *Ω*_arag_.

#### 3.2.3 Changing probability of extreme diurnal cycles of the CO_2_ system

**FIGURE 5:**
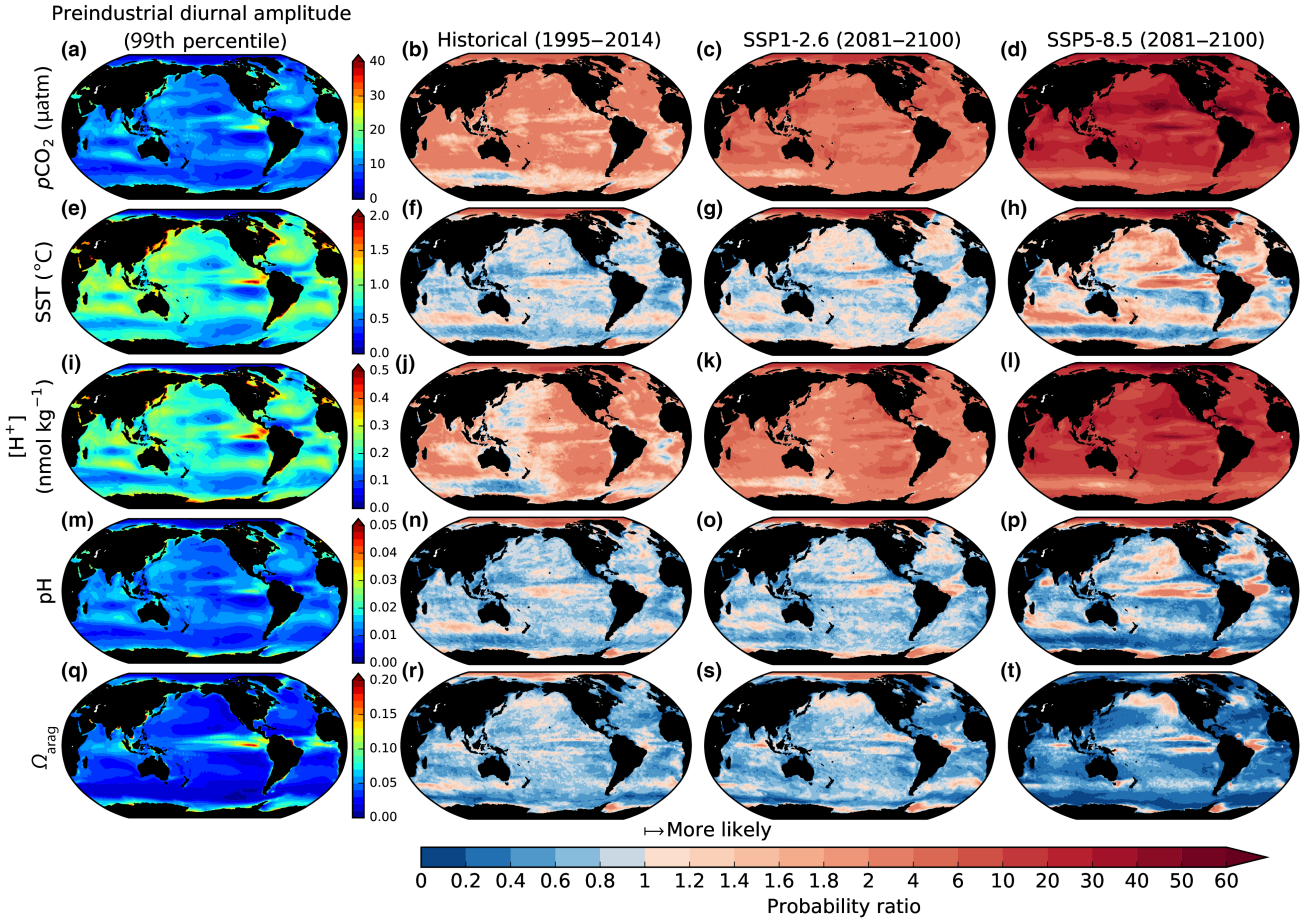
Extreme diurnal amplitudes of *p*CO_2_, SST, [H^+^], pH, and *Ω*_arag_ under preindustrial conditions and their probability ratio in historical and SSP simulations. The 99th percentile of *p*CO_2_, SST, [H^+^], pH, and *Ω*_arag_ diurnal amplitudes in the preindustrial control and the probability ratio in the last 20 years of the historical, SSP1‐2.6, and SSP5‐8.5 simulations. A probability ratio of 10 represents a 10‐fold increase in the probability of a preindustrial extreme diurnal amplitude (a 1 in 100‐day event becomes a 1 in 10‐day event).

The peak‐to‐peak amplitude of extreme diurnal cycles (99th and 99.9th percentile days) under unperturbed preindustrial conditions were characterized using 245 years of 3‐h model output. Simulated 99th percentile diurnal cycles, which by definition are 1 in 100‐day events, correspond to global mean diurnal amplitudes for SST, *p*CO_2_, [H^+^], *Ω*_arag_, and pH of 0.9°C, 10 μatm, 0.2 nmol kg^−1^, 0.04 and 0.01 units, respectively (Figure 5). These area‐weighted, global mean values mask regional variability with enhanced magnitudes of extreme diurnal cycles particularly evident in the tropics and coastal waters and attenuated amplitudes typical of subtropical gyres, the Arctic and Southern Ocean.

By the last 20 years of the historical simulation, there is an increase in the probability of extreme *p*CO_2_ diurnal amplitudes, with a global mean Pr of 2.2 indicating approximately a doubling in the likelihood of 99th percentile preindustrial amplitudes. In the final 20 years of SSP1‐2.6, the Pr increases to 4.3, while under SSP5‐8.5, it increases to 22 (2081–2100 mean). Thus, 1 in 100‐day *p*CO_2_ diurnal amplitudes in the preindustrial become 4.3 times more likely (approximately 1 in 23‐day events) under SSP1‐2.6 and 22 times more likely (approximately 1 in 5‐day events) under SSP5‐8.5.

In the final 20 years of the historical simulation, the global mean Pr for [H^+^] is 1.8, but in the last 20 years of this century, it rises to 3.3 under SSP1‐2.6 and to 15 under SSP5‐8.5 (Figure 5). Global increases in Pr for [H^+^] diurnal amplitudes are thus similar to those of *p*CO_2_ albeit slightly reduced. This similarity is unsurprising given the near‐linear relationship between *p*CO_2_ and [H^+^] on annual timescales (Orr, 2011) and the similar projected amplification of their seasonal and diurnal cycles.

Decreases in the Pr of pH occur despite large increases in the Pr of [H^+^] diurnal amplitudes because of the logarithmic scale of pH and the generally declining annual mean pH over the historical period and SSPs (Fassbender et al., 2021; Kwiatkowski et al., 2020; Kwiatkowski & Orr, 2018).The global mean Pr of pH decreases to 0.84 toward the end of the historical simulation, then rises by the end of the century to 0.92 under SSP1‐2.6 and to 0.95 under SSP5‐8.5. These limited changes in Pr reflect compensation between regional increases and decreases that are highly correlated with changes in the Pr of SST. In particular, increases in the Pr of pH are projected in the Arctic Ocean and decreases in the Southern Ocean.

In contrast to *p*CO_2_ and [H^+^], changes to the probability of extreme diurnal amplitudes of SST, *Ω*_arag_, and pH are more limited, with regions of both enhanced and reduced probability. The global mean Pr of the 99th percentile of SST diurnal amplitudes is 1.0 (unchanged) in the last 20 years of the historical, and by 2081–2100 that rises to 1.2 under SSP1‐2.6 and 1.7 under SSP5‐8.5. These global changes are dominated by high values in the Arctic Ocean where the Pr can exceed 30, particularly in areas of sea ice cover with minimal diurnal SST variability under preindustrial conditions. The reductions in sea ice in these regions during the historical and SSP simulations enhance diurnal variability and result in large increases in the Pr. Such findings are consistent with studies projecting dramatic amplification of SST seasonal cycles in the Arctic Ocean as a consequence of reduced sea ice cover (Alexander et al., 2018; Carton et al., 2015; Kwiatkowski et al., 2020). In contrast to the Arctic, the Pr of 99th percentile SST diurnal amplitudes generally remains below 2 in the rest of the global ocean under both SSPs, with values less than 1 (reduced probability of extremes) in regions such as the Southern Ocean.

The Pr of 99th percentile *Ω*_arag_ diurnal amplitudes declines under the influence of anthropogenic climate change. By the last 20 years of the historical simulation, the global mean Pr reaches 0.73, and declines to 0.71 under SSP1‐2.6 and 0.48 under SSP5‐8.5. As such, 1 in 100‐day diurnal amplitudes of *Ω*_arag_ in the preindustrial simulation become approximately 1 in 200‐day events by the end of SSP5‐8.5 (although mean state *Ω*_arag_ is much lower). These reductions in the probability of *Ω*_arag_ extreme diurnal amplitudes are broadly consistent across the global ocean with only a few isolated regions such as in the Arctic exhibiting limited increases in Pr values during historical and SSP simulations.

Simulated 99.9th percentile diurnal amplitudes, or 1 in 1000‐day events, correspond to *p*CO_2_, SST, [H^+^], *Ω*_arag_, and pH global mean diurnal amplitudes of 19 μatm, 1.4°C, 0.4 nmol kg^−1^, 0.11 and 0.02 units, respectively, in the preindustrial control (Figure S8). Projected increases in the likelihood of these 1 in 1000‐day events in the preindustrial baseline can be even greater than those for the 1 in 100‐day events. In 2081–2100 under SSP‐8.5, the global mean Pr of *p*CO_2_ is 76 and the Pr of [H^+^] is 49. Thus, formerly 1 in 1000‐day extremes for *p*CO_2_ and [H^+^] diurnal amplitudes are projected to become, on average, 1 in 13‐day and 1 in 20‐day events, respectively. The Pr of 99.9th percentile diurnal amplitudes of SST are also enhanced relative to 99th percentile values with the global mean Pr rising to 1.8 under SSP1‐2.6 and 3.3 under SSP5‐8.5 (compared to 1.2 and 1.7 for 99th percentile values under SSP1‐2.6 and SSP5‐8.5, respectively; Figure S8). Conversely, the Pr values of pH and *Ω*_arag_ are broadly consistent between 99th and 99.9th percentile diurnal amplitudes.

#### 3.2.4 Contribution of diurnal variability to absolute extremes

**FIGURE 6:**
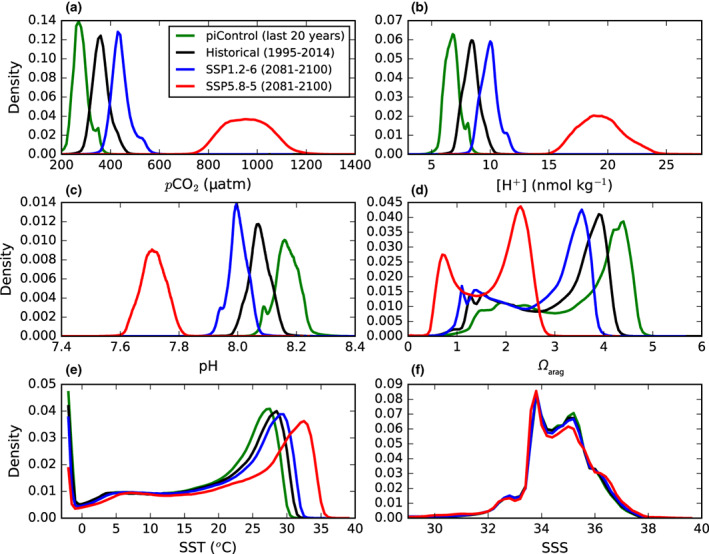
The probability density of (a) *p*CO_2_, (b) [H^+^], (c) pH, (d) *Ω*_arag_, (e) temperature, and (f) salinity in the simulated surface ocean. Distributions are computed from area‐weighted binned monthly model outputs in the final 20 years of the preindustrial, historical, SSP1‐2.6, and SSP5‐8.5 simulations.

Probability density functions derived from binned monthly model output highlight how absolute physical and carbonate chemistry conditions change in the surface ocean over historical and future simulations (Figure 6). The distribution of global surface ocean salinity is broadly consistent across the final 20 years of the preindustrial (34.4 ± 1.9; mean ± 1*σ*), historical (34.4 ± 1.8), SSP1‐2.6 (34.5 ± 1.7), and SSP5‐8.5 (34.5 ± 1.6) simulations. Relative to SST in the preindustrial (17.6 ± 9.9°C), the distribution of SST exhibits increases in mean values in the historical (18.4 ± 10.1°C), SSP1‐2.6 (19.3 ± 10.1°C), and SSP5‐8.5 (21.9 ± 10.4°C) simulations; however, the standard deviations of the distributions remain similar. In contrast, the distributions of CO_2_ system variables respond in terms of both the mean and standard deviation. For *p*CO_2_, [H^+^], and pH, end‐of‐century distributions under SSP5‐8.5 (957 ± 95 μatm, 19 ± 1.9 nmol kg^−1^ and 7.7 ± 0.04, respectively) are distinct from those in the final 20 years of the preindustrial (280 ± 33 μatm, 6.8 ± 0.7 nmol kg^−1^, 8.2 ± 0.04), historical (362 ± 36 μatm, 8.4 ± 0.8 nmol kg^−1^, 8.1 ± 0.04), and SSP1‐2.6 (443 ± 41 μatm, 10.0 ± 0.8 nmol kg^−1^, 8.0 ± 0.04) simulations. There is effectively no overlap in conditions. Furthermore, while the standard deviation of surface temperature and salinity is broadly constant across simulations, there is more than a twofold increase in the standard deviation of *p*CO_2_ and [H^+^] under SSP5‐8.5.

Although the diurnal variability of the surface ocean CO_2_ system is simulated to change dramatically, the contribution of this variability to extremes in absolute values is generally minimal. Simulated and observed surface ocean 99th percentile *p*CO_2_ values are well captured by monthly model outputs (Figure S9; Table S2). The use of daily, instead of monthly, model output typically increases 99th percentile *p*CO_2_ values by <7 μatm (<2%), although increases can be 10–15 μatm in certain coastal regions. In contrast, the use of 3‐h, instead of daily, model output generally has no impact on 99th percentile *p*CO_2_ values, with the exception of isolated parts of the Arctic Ocean and Southern Ocean.

## 4 DISCUSSION

### 4.1 Drivers of increasing *p*CO_2_ diurnal variability

**FIGURE 7:**
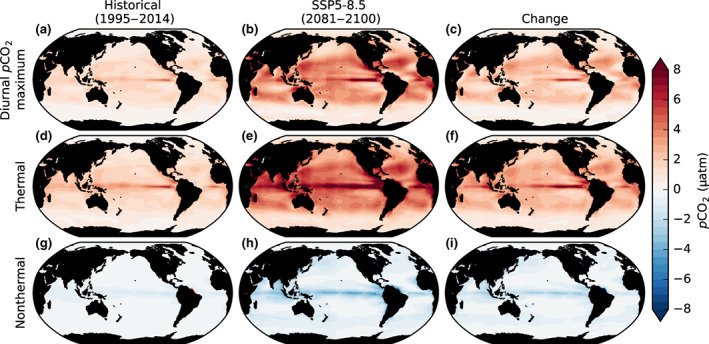
The thermal and nonthermal drivers of diurnal *p*CO_2_ anomaly maxima in the present and future ocean. The *p*CO_2_ diurnal anomaly maxima for (a), the historical (1995–2014); (b), SSP5‐8.5 (2081–2100); and (c), the change between these periods. The thermal (d–f) and nonthermal (g–i) contributions to *p*CO_2_ diurnal maxima are derived from Taylor expansions of the mean diurnal cycle of *p*CO_2_ anomalies for each grid cell. The *p*CO_2_ diurnal anomaly maxima are computed from the mean diurnal cycle of *p*CO_2_, relative to the local mean *p*CO_2_, in each grid cell for each 20‐year period.

Around the end of the historical simulation (1995–2014), maximum diurnal *p*CO_2_ anomalies, relative to daily mean values, are thermally driven and in some regions attenuated slightly by a coincident nonthermal contribution (Figure 7). As previously discussed, this thermal dominance agrees broadly with open ocean observations at time‐series stations, where diurnal *p*CO_2_ maxima typically occur around midday as a consequence of surface heat fluxes producing thermal maxima (Bates et al., 1998; Torres et al., 2021). The high thermal sensitivity of *p*CO_2_ is largely a consequence of the effect of temperature on CO_2_ solubility; *p*CO_2_ also increases because of the effect of temperature on the CO_2_ system dissociation constants (*K*_1_ and *K*_2_), but that effect is secondary. The nonthermal contribution is consistently negative as it is primarily driven by NPP in the model. At the time of diurnal *p*CO_2_ maxima in the early afternoon, SST anomalies are at their maxima and photosynthetic active radiation is high. This is therefore consistently a time of the day when simulated NPP is positive and acts to reduce the concentration of DIC, and consequently *p*CO_2_. The enhanced negative contribution in equatorial waters is due to this being a region of high phytoplankton productivity (Figure S2).

During the SSP5‐8.5 simulation, diurnal *p*CO_2_ maxima increase by up to 4 μatm in the low and mid‐latitude oceans. These increases are driven by an enhanced thermal contribution to *p*CO_2_ maxima and despite a coincident but lesser enhancement in the attenuating nonthermal contribution (Figure 7). As such, throughout SSP5‐8.5, the thermal dominance is reinforced for *p*CO_2_ maxima only being partly offset by nonthermal processes. Outside of the Arctic Ocean, the diurnal amplitude of SST changes little throughout SSP5‐8.5. Thus, the continued dominance of the thermal contribution occurs because the sensitivity of *p*CO_2_ to temperature (*∂p*CO_2_/*∂T*) increases as ocean carbon content increases. Although the nonthermal sensitivities *∂p*CO_2_/*∂C*_T_ and ∂*p*CO_2_/∂*A*_T_ increase more (Gallego et al., 2018), the absolute contribution of the nonthermal component remains small. The enhanced sensitivity of *p*CO_2_ to variations in temperature, DIC, and alkalinity, or enhanced *p*CO_2_ buffer factors (Egleston et al., 2010), is nonlinear and occurs in response to the rising DIC concentration in the surface ocean with a greater fraction of that DIC in the form of HCO_3_^−^ and CO_2_ (aq) and a reduced fraction in the form of ${\mathrm{CO}}_{3}^{2-}$. Similar dominance of the thermal component of *p*CO_2_ variability combined with increases in ∂*p*CO_2_/∂T has previously been shown to explain projected amplification of the *p*CO_2_ seasonal cycle by CMIP5 models in the same subtropical and equatorial regions (Gallego et al., 2018).

The simulated magnitude of anomalies relative to the daily mean are smaller for diurnal *p*CO_2_ minima than for diurnal *p*CO_2_ maxima (compare Figure 7 to Figure S10) because the amplitude of nocturnal SST minima is less than daytime SST maxima. In contrast, the attenuating nonthermal contribution to diurnal *p*CO_2_ anomalies is similar in absolute magnitude for both *p*CO_2_ minima and maxima. The magnitude of the minima of diurnal *p*CO_2_ anomalies is therefore also amplified during SSP5‐8.5 but to a lesser extent than *p*CO_2_ maxima (around 2 μatm compared to 3 μatm in the low and mid‐latitudes) as a result of the reduced dominance of the thermal contribution relative to the nonthermal contribution.

### 4.2 Diurnal variability and absolute extremes

Extreme absolute values of surface ocean *p*CO_2_ simulated by the model are well represented when using only monthly model output. Using finer temporal resolution, daily means and indeed 3‐h model output have limited effect on the magnitude of extreme absolute *p*CO_2_ (Figure S9). This finding indicates that the modeled seasonal *p*CO_2_ variability is considerably larger than daily and diurnal variability and that seasonal *p*CO_2_ maxima and minima may not necessarily coincide with periods where daily or diurnal *p*CO_2_ variability is highest. However, given that the model underestimates *p*CO_2_ diurnal variability and notably extreme diurnal variability (Figure 2), it is also likely to underestimate the contribution of diurnal variability to extreme absolute values of surface ocean *p*CO_2_. Improved representation of diurnal variability in models should therefore remain a long‐term development focus.

Whether or not diurnal variability contributes greatly to absolute extremes, the rate of change of CO_2_ system variables on diurnal timescales typically far exceeds that on seasonal timescales and may well affect ecosystems exposed to temporally dynamic chemistry regimes (Kroeker et al., 2020). The sensitivity of ecosystems to projected increases in CO_2_ system diurnal variability may therefore depend not only on their sensitivity to absolute thresholds but also the rate of change of the CO_2_ system.

### 4.3 Potential ecosystem impacts

#### 4.3.1 Insights from diurnal CO_2_ system manipulation studies

Experimental studies on marine organisms suggest that our projected increases in the diurnal variability of *p*CO_2_ and [H^+^] will have diverse ecosystem impacts. Diurnal variability of *p*CO_2_ can increase organism metabolic costs (Mangan et al., 2017) and decrease the growth and calcification of certain species (Cornwall et al., 2013; Jiang et al., 2019). However, there is also evidence that diurnal CO_2_ system variability can increase the growth rates of species (Dufault et al., 2012) and mitigate other ocean acidification impacts (Laubenstein et al., 2020). It is pertinent, however, that most aquaria studies to date have compared organism responses between static and diurnally variable CO_2_ system regimes and therefore not assessed the influence of augmented diurnal regimes. Furthermore, no aquaria study has assessed the impact of augmented CO_2_ system diurnal variability alongside coincident changes in both mean background conditions and seasonal variability.

#### 4.3.2 Organism exposure to modified diurnal variability

When assessing potential ecosystem impacts, a further consideration is the extent of organism exposure to the modified CO_2_ system diurnal variability. Our projections are, for computational reasons, limited to the upper meter of the global ocean. However, few organisms are confined to the surface ocean, and thus, projections of diurnal CO_2_ system variability in subsurface waters are also needed. Furthermore, planktonic organisms are advected with water masses and therefore fixed‐point diurnal variability may not be indicative of the variability such organisms experience within a given water mass (Kroeker et al., 2020). Finally, while our projections of changes in CO_2_ system diurnal variability may be indicative of the bulk seawater conditions that shallow, sessile organisms are exposed to, our ocean biogeochemical model is designed for open ocean applications. Extensive diurnal variability of the CO_2_ system, particularly in coastal waters and boundary layers, is often a consequence of productive ecosystems such as kelp forests (Murie & Bourdeau, 2020), seagrasses (Berg et al., 2019), and coral reefs (Drupp et al., 2013). As these ecosystems are not represented in NEMO‐PISCES‐QUOTA, the influence of climate change on them and in turn their influence on diurnal variability of the ocean CO_2_ system is currently unaccounted for.

## 5 CONCLUSIONS

Here, we present results from simulations of a global ocean biogeochemical model that resolves both physically and biologically driven diurnal cycles of the ocean CO_2_ system. The model was forced with 3‐h atmospheric outputs of an Earth system model to assess how the diurnal variability of the surface ocean CO_2_ system is influenced by rising atmospheric CO_2_ and associated climate change. The phasing of simulated present‐day diurnal cycles of surface ocean *p*CO_2_, pH, *Ω*_arag_, and temperature compares well to time‐series observations in the open ocean, lending confidence to the historical and future model projections. Relative to 1850–1869, the simulated global mean diurnal amplitude of *p*CO_2_ and [H^+^] increases by 4.8 μatm (+226%) and 0.07 nmol kg^−1^ (+170%), respectively, by 2081–2100 in the high‐emission scenario SSP5‐8.5, with most of that amplification occurring in the second half of the 21st century. Over the same period, the global mean diurnal amplitude of pH and *Ω*_arag_ decreases by 0.0001 (−4%) and 0.0025 (−22%), respectively. Projected changes are smaller under the high‐mitigation scenario SSP1‐2.6, with the increase in the diurnal amplitudes of *p*CO_2_ and [H^+^] reaching 1.2 μatm (+55%) and 0.018 nmol kg^−1^ (+45%), respectively, and that of *Ω*_arag_ declining by 0.0006 (−5%) while for pH, there is no change. The principal driver of amplified *p*CO_2_ diurnal variability is the enhanced sensitivity of ocean *p*CO_2_ to diurnal variations in temperature as the anthropogenic carbon content of the ocean increases. In our simulations, diurnal variability has very little impact on absolute extremes of the surface ocean CO_2_ system over most of the global ocean. However, the occurrence of extreme diurnal amplitudes of the CO_2_ system substantially changes, with 30‐ to 60‐fold increases in the frequency of preindustrial extreme diurnal amplitudes of *p*CO_2_ and [H^+^] under high 21st‐century emissions. Our projections suggest that the metabolic cost that diurnal variability of the CO_2_ system imposes on organisms will increase this century, with potential consequences for marine ecosystem health.

## AUTHOR CONTRIBUTIONS

Lester Kwiatkowski, James C. Orr, and Olivier Aumont conceived the study. Olivier Torres adapted the model code and performed the simulations and analysis. Lester Kwiatkowski led the writing of the manuscript with all co‐authors contributing.

## CONFLICT OF INTEREST

The authors declare that they have no conflict of interest.

## DISCLAIMER

This article reflects only the authors' views; the funding agencies and their executive agencies are not responsible for any use that may be made of the information that the article contains.

## Supporting information


Appendix S1.

## Data Availability

The data that support the findings of this study are openly available in Zenodo at: https://doi.org/10.5281/zenodo.6772019.
