# Supplementary material for: Modified future diurnal variability of the global surface ocean CO2 system
Source: Glob Chang Biol. 2022 Nov 20;29(4):982–97. doi: 10.1111/gcb.16514 (PMC10098810; doi:10.1111/gcb.16514)
Supplement: Supplementary file 1 — Appendix S1. [file GCB-29-982-s001.pdf]

Modified future diurnal variability of the global surface ocean CO_2 system: Supplementary material

Lester Kwiatkowski[1], Olivier Torres[2], Olivier Aumont[1] and James C. Orr[3]

[1]LOCEAN Laboratory, Sorbonne Université-CNRS-IRD-MNHN, Paris, 75005, France
[2]LMD-IPSL, CNRS, Ecole Normale Supérieure / PSL Res. Univ, Ecole Polytechnique, Sorbonne Université, Paris, 75005, France
[3]Laboratoire des Sciences du Climat et de l'Environnement, LSCE-IPSL, CEA-CNRS-UVSQ, Université Paris Saclay, Gif-sur-Yvette, France

*Kwiatkowski and Torres should be considered joint first author

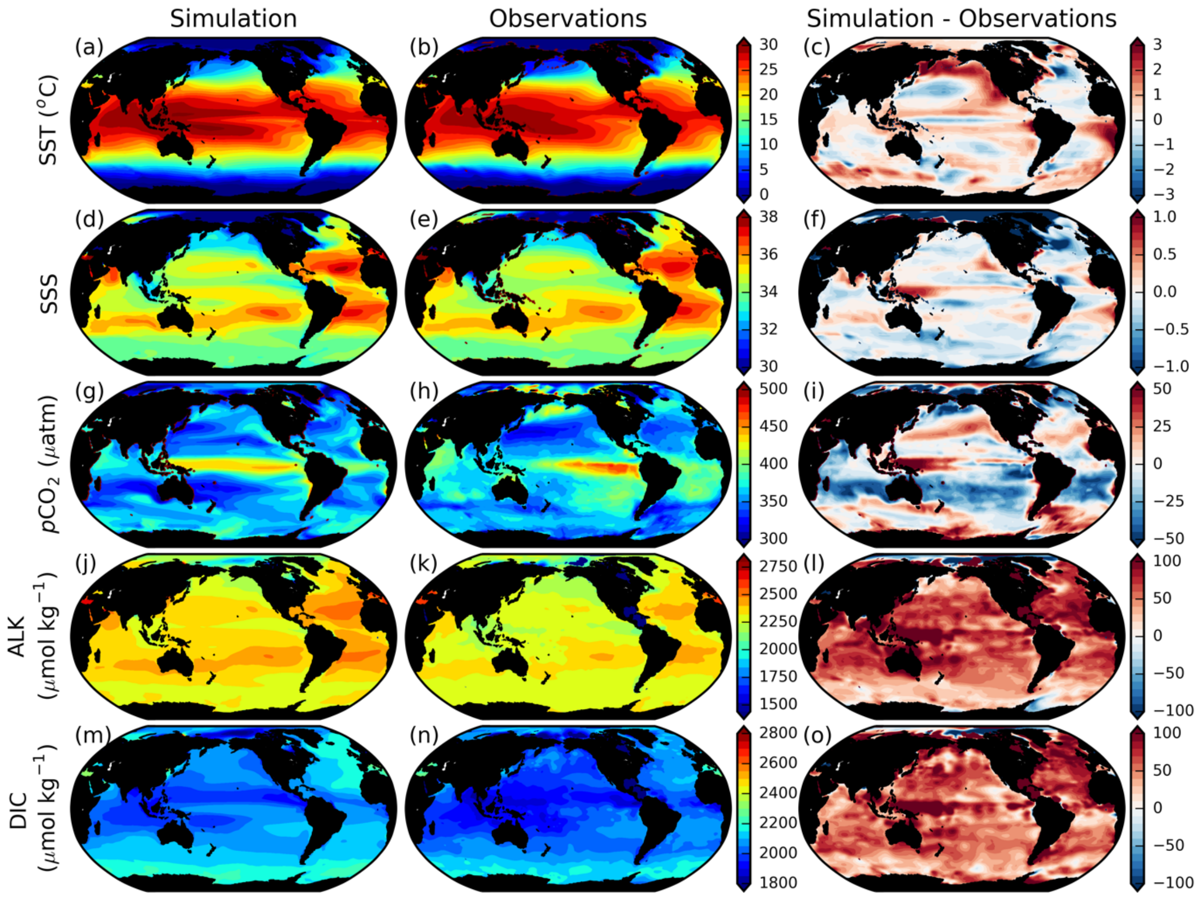

Figure S1. Comparison of observed and simulated annual mean sea surface temperature (SST), salinity (SSS), *p*CO_2, alkalinity and DIC. Temperature and salinity observations are from the World Ocean Atlas 2018 (Locarnini et al., 2018; Zweng et al., 2019), *p*CO_2 is the neural network derived observational product of Landschützer et al., 2020, and DIC and alkalinity observations are from GLODAP version 2 (Lauvset et al., 2016). Model annual means are averaged over 1998-2007 with the exception of *p*CO_2 which is averaged over 1988-2014.

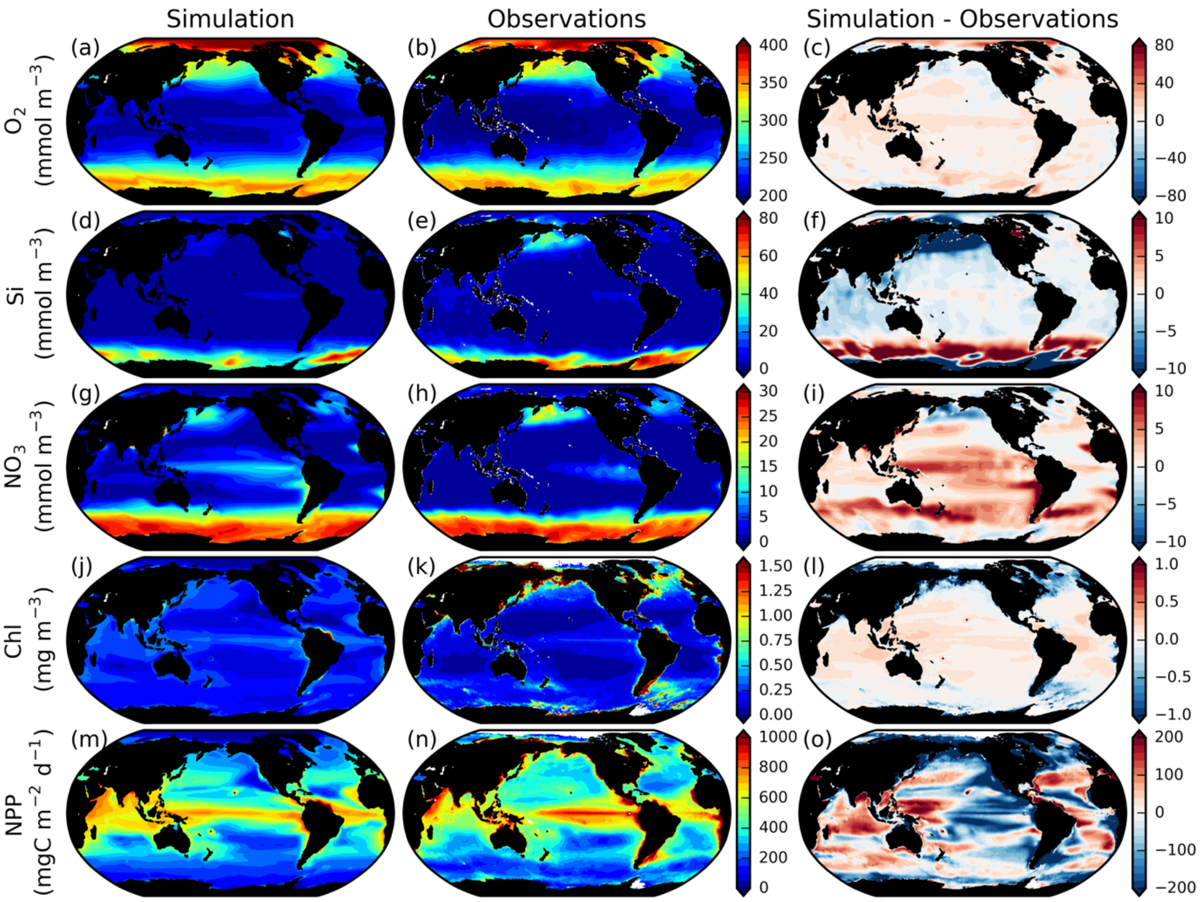

Figure S2. Comparison of observed and simulated annual mean surface ocean oxygen, silicate, nitrate, chlorophyll a concentrations and depth integrated primary production (NPP). Oxygen, Si and NO_3 observations are from the World Ocean Atlas 2018 (Garcia et al., 2019a, 2019b), chlorophyll a concentration is from NASA's Moderate-resolution Imaging Spectroradiometer (MODIS, Hu et al., 2012; Werdell & Bailey, 2005). NPP is the Carbon-based Productivity Model observational product (Behrenfeld et al., 2005; Westberry et al., 2008). Model annual means are averaged over 1998-2007.

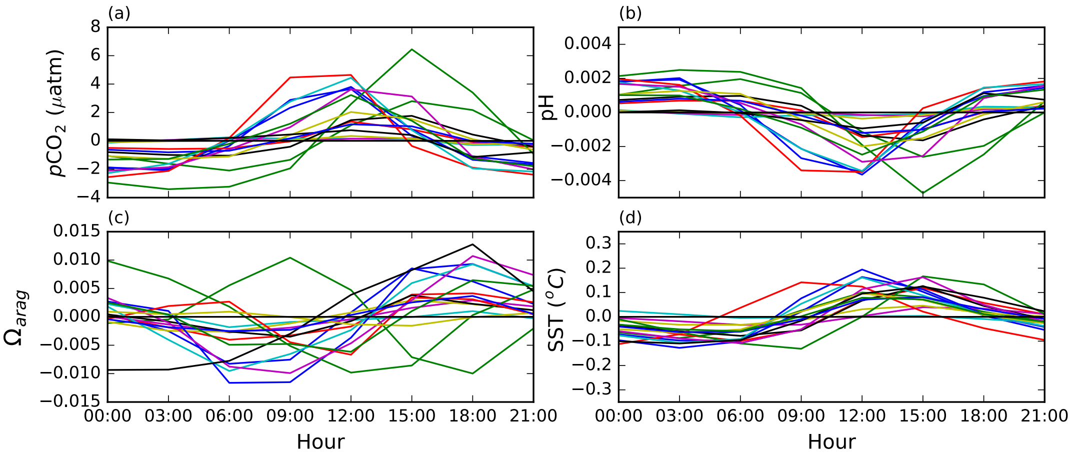

Figure S3. Observed diurnal cycles of the CO_2 system in the surface open ocean. Mean observed diurnal cycles of pCO$_2$, pH, Ω_{arag} and temperature at each of the 15 open ocean stations (Sutton et al., 2019; Torres et al., 2021).

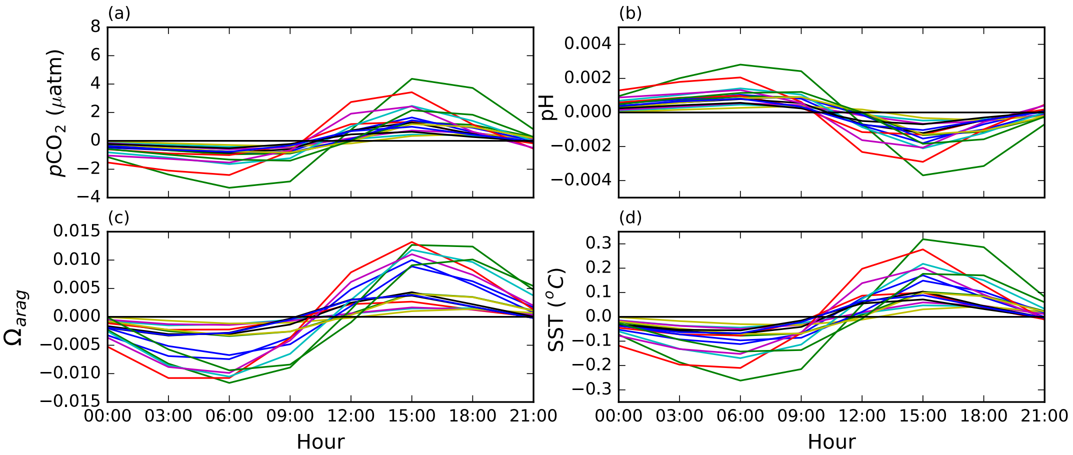

Figure S4. Simulated diurnal cycles of the CO_2 system in the surface open ocean. Mean simulated diurnal cycles of $p$$CO_2$, pH, Ω_{arag} and temperature at each of the 15 open ocean stations shown in Figure S3, with model outputs extracted from the nearest grid cell to each observational station over the period 1995-2014.

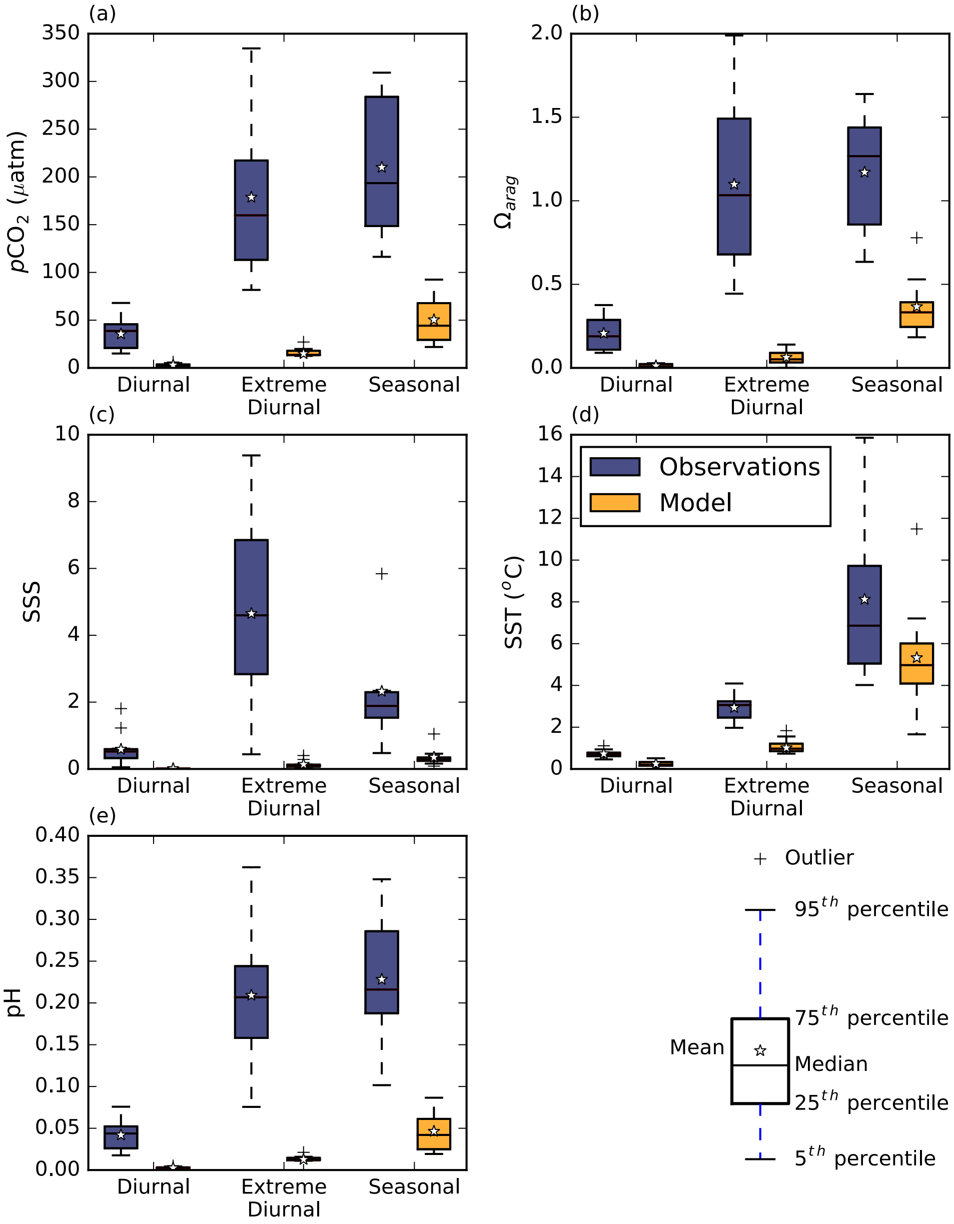

Figure S5. Evaluation of the amplitude of simulated diurnal, extreme diurnal and seasonal cycles of the CO_2 system in the surface coastal ocean. The modelled (orange) coastal ocean diurnal, extreme diurnal (99th percentile) and seasonal cycles of pCO_2, Ω_{arag}, salinity, temperature and pH compared to fixed timeseries observations (blue; Sutton et al., 2019; Torres et al., 2021). Boxplots are produced from the station mean amplitude of cycles across 11 coastal ocean observational stations with model outputs extracted from the nearest grid cell to each observational station over the period 1995-2014.

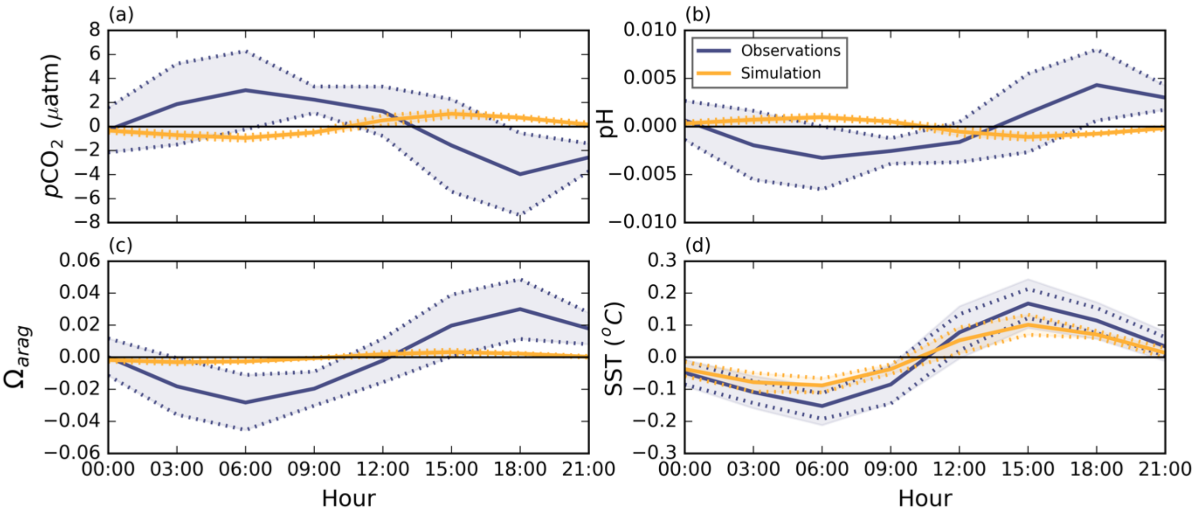

Figure S6. Evaluation of simulated diurnal cycles of the CO_2 system in the coastal ocean against observations. Mean simulated (orange) and observed (blue; Sutton et al., 2019; Torres et al., 2021) diurnal cycles of pCO_2, pH, Ω_{arag} and SST across 11 coastal ocean station locations, with model outputs extracted from the nearest grid cell to each observational location over the period 1995-2014.

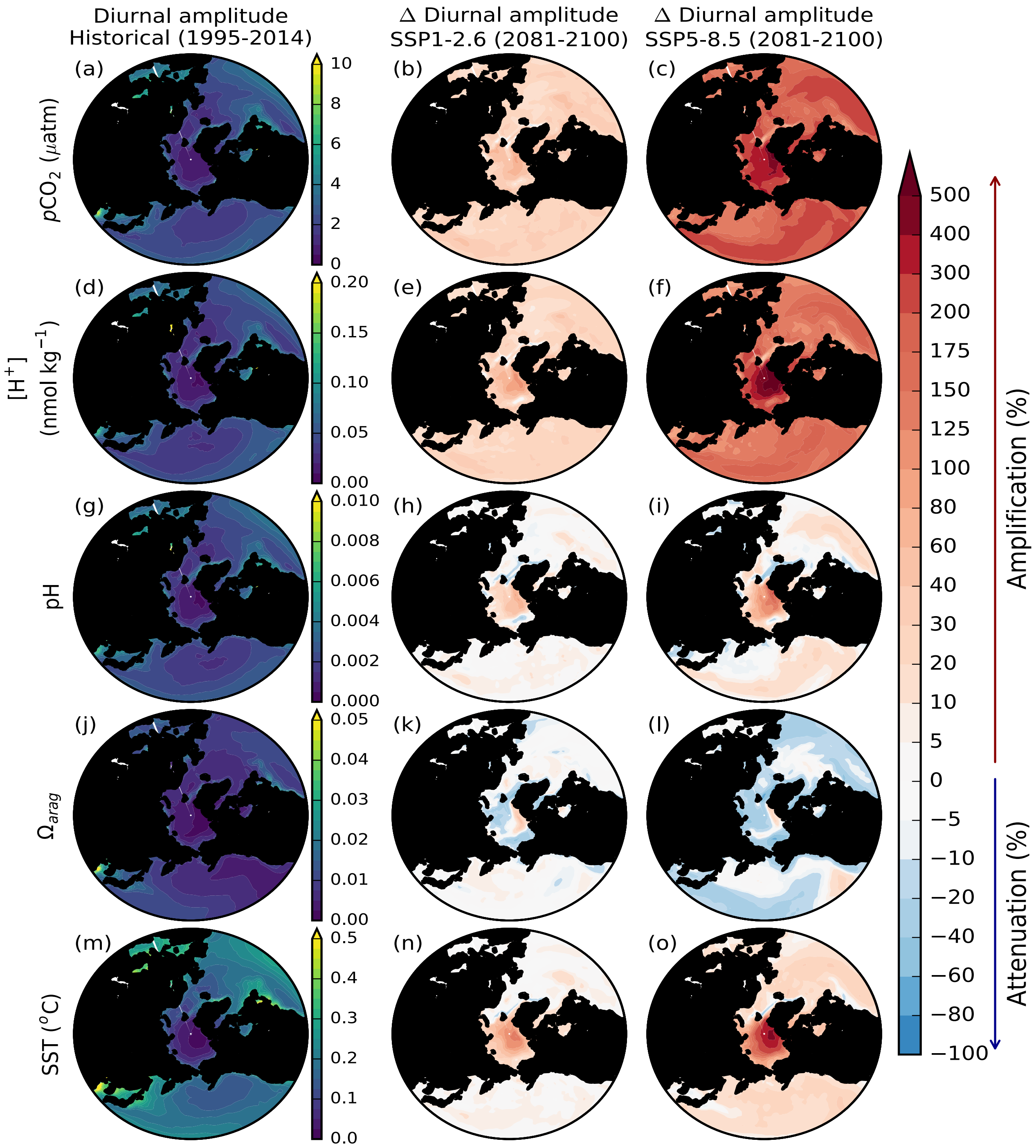

Figure S7. The simulated Arctic diurnal amplitudes of pCO$_2$, [H$^+$], pH, Ω$_{arag}$ and SST and their amplification/attenuation under SSP1-2.6 and SSP5-8.5. A polar projection of Figure 4 showing the simulated mean modern (1995-2014) diurnal amplitudes of pCO$_2$, [H$^+$], pH, Ω$_{arag}$ and SST and their change in 2081-2100 under SSP1-2.6 and SSP5-8.5 relative to the historical period.

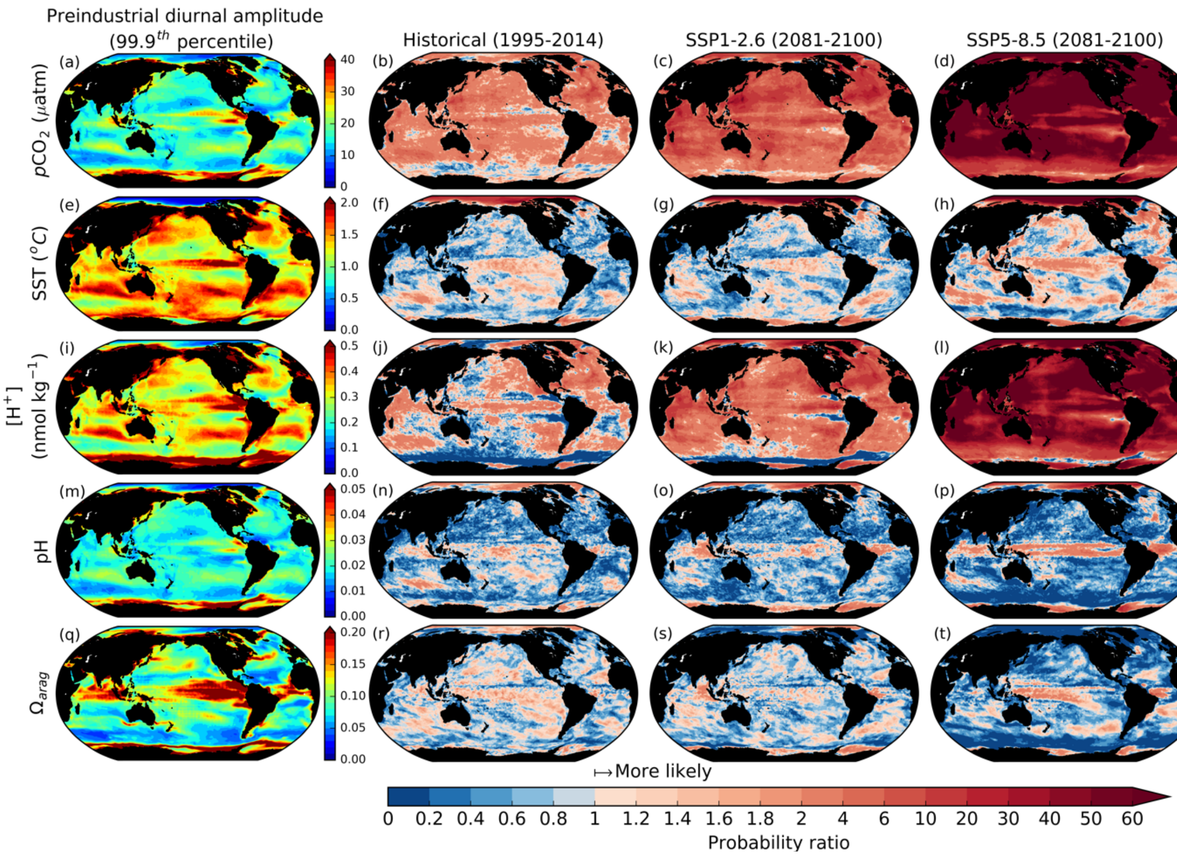

Figure S8. Extreme diurnal amplitudes of pCO$_2$, SST, Ω_{arag} and pH under preindustrial conditions and the change in their probability under climate change. The 99.9th percentile of pCO$_2$, SST, Ω_{arag} and pH diurnal amplitudes in the preindustrial control and the change in the probability of these thresholds being exceeded in the last 20 years of the historical, SSP1-2.6 and SSP5-8.5.

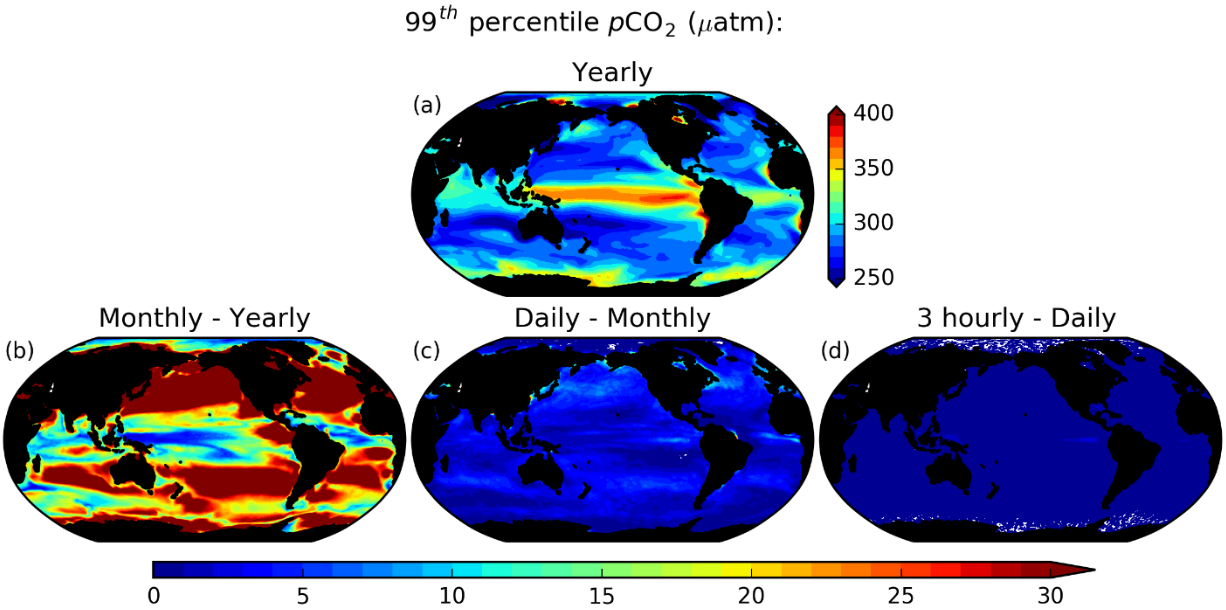

Figure S9. The 99th percentile of simulated pCO_2 in the surface ocean using model output at varying temporal resolution. The a, 99th percentile of pCO_2 in the surface ocean as determined using annual model output and the increase in 99th percentile pCO_2 if computed from b, monthly, c, daily and d, 3-hourly model output. All model output corresponds to the same multi-centennial period of the preindustrial control.

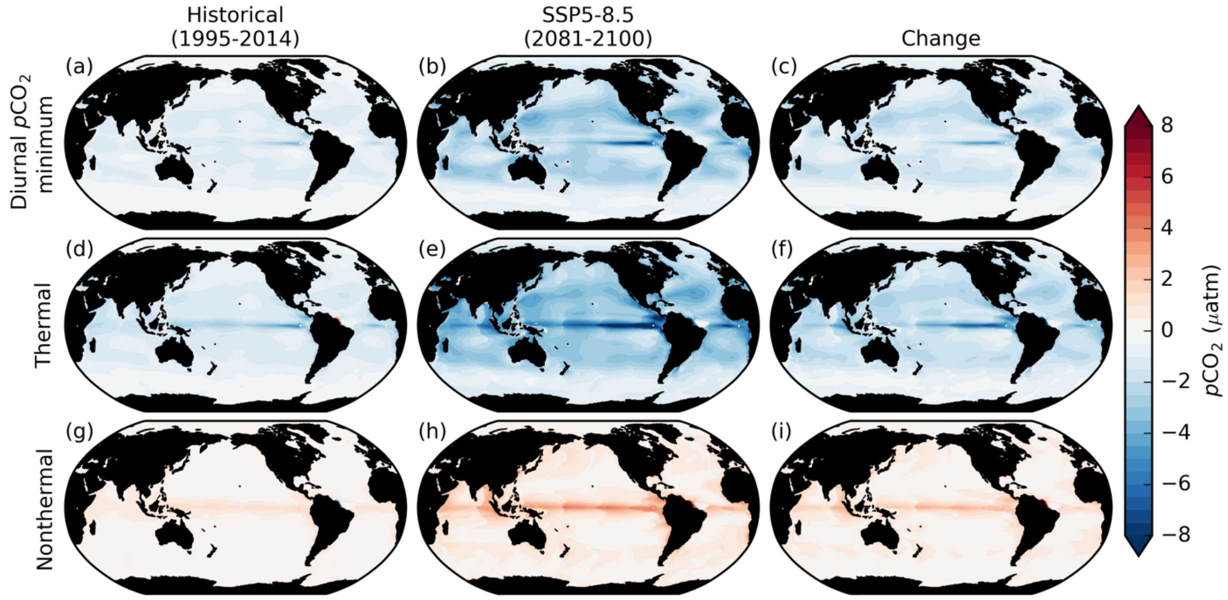

Figure S10. The thermal and nonthermal drivers of diurnal *p*CO_2 minima in the present and future ocean. The *p*CO_2 diurnal anomaly minima for a, the historical (1995-2014), b, SSP5-8.5 (2081-2100), and c, the change between these periods. The thermal (d-f) and nonthermal (g-i) contribution to *p*CO_2 diurnal minima are derived from Taylor expansions of the mean diurnal cycle of *p*CO_2 anomalies for each grid cell and period. *p*CO_2 diurnal anomaly minima are computed from the mean diurnal cycle of *p*CO_2, relative to the local mean *p*CO_2, in each grid cell for each twenty-year period.

Table S1. Global area-weighted mean values of the simulated CO_2 system. The global annual mean, seasonal amplitude and diurnal amplitude of pCO_2, [H^+], pH and Ω_{arag} in the first and last twenty years of the historical simulation and the last twenty years of the SSP simulations.

Simulation	Period	pCO_2 (µatm)			[H^+] (nmol kg^{-1})			pH			Ω_{arag}		
		Annual mean	Seasonal amplitude	Diurnal amplitude	Annual mean	Seasonal amplitude	Diurnal amplitude	Annual mean	Seasonal amplitude	Diurnal amplitude	Annual mean	Seasonal amplitude	Diurnal amplitude
Historical	1850-1869	286	47	2.11	6.65	0.94	0.040	8.16	0.059	0.00252	3.43	0.394	0.0113
	1995-2014	367	57	2.66	8.18	1.09	0.049	8.07	0.056	0.00248	3.02	0.382	0.0109
SSP1-2.6	2081-2100	448	67	3.27	9.68	1.24	0.058	8.00	0.054	0.00248	2.72	0.396	0.0107
SSP5-8.5	2081-2100	963	117	6.87	18.8	1.92	0.108	7.71	0.043	0.00242	1.74	0.354	0.0088

Table S2. The 99th percentile of observed pCO_2 in the surface ocean at stations with at least 100 months of observations. The 99th percentile of pCO_2 in the surface ocean as determined using monthly mean station observations (Sutton et al., 2019; Torres et al., 2021) and the increase in 99th percentile pCO_2 if computed from daily and 3-hourly observations.

**Station name**	**Station Type**	**Months**	**Days**	**Monthly 99th percentile pCO_2 (μatm)**	**Daily – monthly 99th percentile pCO_2 (μatm)**	**3 hourly – daily 99th percentile pCO_2 (μatm)**
STRATUS	Open	103	3092	440	8	1
TAO 125W	Open	154	4658	545	18	3
TAO 140W	Open	131	3956	511	16	9
WHOTS	Open	128	3861	403	1	2
CAPE ELIZABETH	Coastal	113	3417	429	22	10
GRAYS REEF	Coastal	112	3377	538	19	3
GULF OF MAINE	Coastal	113	3401	481	17	7

References

Behrenfeld, M. J., Boss, E., Siegel, D. A., & Shea, D. M. (2005). Carbon-based ocean productivity and phytoplankton physiology from space. *Global Biogeochemical Cycles*, *19*(1), GB1006. https://doi.org/10.1029/2004GB002299

Garcia, H., Weathers, K., Paver, C., Smolyar, I., Boyer, T., Locarnini, M., Zweng, M., Mishonov, A., Baranova, O., Seidov, D., & Reagan, J. (2019a). *World Ocean Atlas 2018. Vol. 4: Dissolved Inorganic Nutrients (phosphate, nitrate and nitrate+nitrite, silicate)*. https://archimer.ifremer.fr/doc/00651/76336/

Garcia, H., Weathers, K., Paver, C., Smolyar, I., Boyer, T., Locarnini, M., Zweng, M., Mishonov, A., Baranova, O., Seidov, D., & Reagan, J. (2019b). *World Ocean Atlas 2018, Volume 3: Dissolved Oxygen, Apparent Oxygen Utilization, and Dissolved Oxygen Saturation.* https://archimer.ifremer.fr/doc/00651/76337/

Hu, C., Lee, Z., & Franz, B. (2012). Chlorophyll aalgorithms for oligotrophic oceans: A novel approach based on three-band reflectance difference. *Journal of Geophysical Research: Oceans*, *117*(C1). https://doi.org/10.1029/2011JC007395

Landschützer, P., Laruelle, G. G., Roobaert, A., & Regnier, P. (2020). A uniform $p\mathrm{CO}_2$ climatology combining open and coastal oceans. *Earth System Science Data*, *12*(4), 2537–2553. https://doi.org/10.5194/essd-12-2537-2020

Lauvset, S. K., Key, R. M., Olsen, A., Heuven, S. van, Velo, A., Lin, X., Schirnick, C., Kozyr, A., Tanhua, T., Hoppema, M., Jutterström, S., Steinfeldt, R., Jeansson, E., Ishii, M., Perez, F. F., Suzuki, T., & Watelet, S. (2016). A new global interior ocean mapped climatology: The 1° × 1° GLODAP version 2. *Earth System Science Data*, *8*(2), 325–340. https://doi.org/10.5194/essd-8-325-2016

Locarnini, M., Mishonov, A., Baranova, O., Boyer, T., Zweng, M., Garcia, H., Reagan, J., Seidov, D., Weathers, K., Paver, C., & Smolyar, I. (2018). *World Ocean Atlas 2018, Volume 1: Temperature*. https://archimer.ifremer.fr/doc/00651/76338/

Sutton, A. J., Feely, R. A., Maenner-Jones, S., Musielwicz, S., Osborne, J., Dietrich, C., Monacci, N., Cross, J., Bott, R., Kozyr, A., Andersson, A. J., Bates, N. R., Cai, W.-J., Cronin, M. F., De Carlo, E. H., Hales, B., Howden, S. D., Lee, C. M., Manzello, D. P., … Weller, R. A. (2019). Autonomous seawater $p\mathrm{CO}_2$ and pH time series from 40 surface buoys and the emergence of anthropogenic trends. *Earth System Science Data*, *11*(1), 421–439. https://doi.org/10.5194/essd-11-421-2019

Torres, O., Kwiatkowski, L., Sutton, A. J., Dorey, N., & Orr, J. C. (2021). Characterizing Mean and Extreme Diurnal Variability of Ocean CO2 System Variables Across Marine Environments. *Geophysical Research Letters*, *48*(5), e2020GL090228. https://doi.org/10.1029/2020GL090228

Werdell, P. J., & Bailey, S. W. (2005). An improved in-situ bio-optical data set for ocean color algorithm development and satellite data product validation. *Remote Sensing of Environment*, *98*(1), 122–140. https://doi.org/10.1016/j.rse.2005.07.001

Westberry, T., Behrenfeld, M. J., Siegel, D. A., & Boss, E. (2008). Carbon-based primary productivity modeling with vertically resolved photoacclimation. *Global Biogeochemical Cycles*, *22*(2). https://doi.org/10.1029/2007GB003078

Zweng, M., Reagan, J., Seidov, D., Boyer, T., Locarnini, M., Garcia, H., Mishonov, A., Baranova, O., Weathers, K., Paver, C., & Smolyar, I. (2019). *World Ocean Atlas 2018, Volume 2: Salinity*. https://archimer.ifremer.fr/doc/00651/76339/
